# AgGaS_2_ and Derivatives: Design, Synthesis, and Optical Properties

**DOI:** 10.3390/nano15020147

**Published:** 2025-01-20

**Authors:** Guansheng Xing, Bing Chen

**Affiliations:** College of Electronic and Optical Engineering and College of Flexible Electronics (Future Technology), Nanjing University of Posts and Telecommunications, Nanjing 210023, China; 1022233819@njupt.edu.cn

**Keywords:** AgGaS_2_, chalcopyrite, nanocrystals, nonlinear optics, optoelectronics

## Abstract

Silver gallium sulfide (AgGaS_2_) is a ternary A^(I)^B^(III)^X^(VI)^_2_-type semiconductor featuring a direct bandgap and high chemical stability. Structurally resembling diamond, AgGaS_2_ has gained considerable attention as a highly promising material for nonlinear optical applications such as second harmonic generation and optical parametric oscillation. In attempts to expand the research scope, on the one hand, AgGaS_2_-derived bulk materials with similar diamond-like configurations have been investigated for the enhancement of nonlinear optics performance, especially the improvement of laser-induced damage thresholds and/or nonlinear coefficients; on the other hand, nanoscale AgGaS_2_ and its derivatives have been synthesized with sizes as low as the exciton Bohr radius for the realization of potential applications in the fields of optoelectronics and lighting. This review article focuses on recent advancements and future opportunities in the design of both bulk and nanocrystalline AgGaS_2_ and its derivatives, covering structural, electronic, and chemical aspects. By delving into the properties of AgGaS_2_ in bulk and nanocrystalline states, this review aims to deepen the understanding of chalcopyrite materials and maximize their utilization in photon conversion and beyond.

## 1. Introduction

In recent years, semiconductors that adopt a chalcopyrite crystal structure and are characterized by the formula A^(I)^B^(III)^X^(VI)^_2_ (A = Cu, Ag; B = Ga, In; X = S, Se or Te) have garnered significant attention due to their attractive applications in nonlinear optical devices, photon detectors, light-emitting diodes, and solar cells. These materials possess several advantages, including low toxicity, high structural adaptability, and suitable bandgaps [1,2,3,4,5,6,7,8,9]. Among the diverse chalcopyrite compounds, AgGaS_2_ stands out, with its transparency window spanning from 0.45 to 13 µm and a substantial bulk bandgap of approximately 2.7 eV. Consequently, AgGaS_2_ exhibits exceptional optical properties, both linear and nonlinear, making it well suited for applications such as photon upconversion, second harmonic generation, and optical parametric oscillation. These capabilities allow AgGaS_2_ to promote photon conversion across a wide spectral range while offering solid-state convenience, reliability, and the ability to handle high-power pulses.

**Figure 1:**
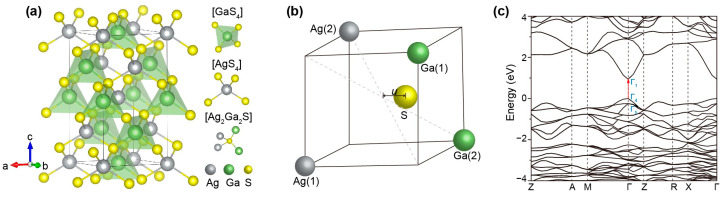
(**a**) Crystal structure of AgGaS_2_ and the [GaS_4_], [AgS_4_], and [Ag_2_Ga_2_S] tetrahedra. (**b**) S^2−^ displacement in chalcopyrite AgGaS_2_. (**c**) Energy levels of chalcopyrite AgGaS_2_ exhibiting a nonzero direct energy gap at the Γ point. Reprinted with permission [12]. Copyright 2022, Wiley-VCH GmbH.

Chalcopyrite AgGaS_2_ belongs to the *I*-42*d* space group, in which each Ag^+^ or Ga^3+^ is coordinated tetrahedrally by four S^2−^ and, correspondingly, each S^2−^ is also tetrahedrally coordinated by two Ag^+^ and two Ga^3+^ cations with different bond lengths (*d*_Ag-S_ ≠ *d*_Ga-S_) to form a tetragonal structure (Figure 1a). Interestingly, the unit cell of titled chalcopyrite AgGaS_2_ can be constructed from two zincblende unit cells aligned in the *c*-direction. The inequality between *d*_Ag-S_ and *d*_Ga-S_ is a direct result of different interaction strengths between Ag^+^/S^2−^ and Ga^3+^/S^2−^ pairs. From the perspective of crystallography, the atomic positions of an ABX_2_-type chalcopyrite unit cell can be defined as A (0, 0, 0), (0, 1/2, 1/2); B (1/2, 1/2, 0); (1/2, 0, 1/4), X (*u*, 1/4, 1/8), (−*u*, 3/4, 1/8), (3/4, *u*, 7/8), (1/4, −*u*, 7/8). Here, *u* is a parameter describing the displacement of the X^2−^ anion from the center of the [A_2_B_2_X] tetrahedron (Figure 1b), which can be defined using the following equation [10]:

(1) $$u=\frac{1}{2}-\sqrt{\frac{c^{2}}{32a^{2}}-\frac{1}{16}}$$

where *a* and *c* are unit cell parameters for the chalcopyrite ABX_2_, and *η* = *c*/2*a* is defined as the geometrical parameter. When considering a standard zincblende structure, it is easy to conclude that *u* equals 1/4. Thus (*u* − 1/4) is regarded as anion displacement, directly related to the near-neighbor cation–anion distances, as shown in the following equations:

(2) $$u-\frac{1}{4}=\frac{d_{AX}^{2}-d_{BX}^{2}}{a^{2}}$$

(3) $$d_{AX}=a\sqrt{u^{2}+\frac{1+\eta^{2}}{16}}\mathrm{and}\;d_{BX}=a\sqrt{{\left(u-\frac{1}{2}\right)}^{2}+\frac{1+\eta^{2}}{16}}$$

Here, *η* = *c*/2*a* can be used to describe the compression of the chalcopyrite unit cell (*η* = 1 for a zincblende unit cell). Experimental measurements have determined the Ag–S and Ga–S distances in AgGaS_2_ to be 2.556 ± 0.001 and 2.276 ± 0.001 Å, respectively [11]. The values of *η* (ranging from 0.89 to 0.93) and *u* (ranging from 0.28 to 0.30) have been documented by Laksari’s and Piasecki’s research groups [10,12]. From the perspective of crystallography, the non-centrosymmetric nature of the tetragonal structure (*η* ≠ 1) is crucial for AgGaS_2_’s exceptional performance in the field of nonlinear optics (NLO), as it enables birefringence and phase matching. Notably, AgGaS_2_ also exhibits superior structural rigidity due to the significant difference in ionic radii between Ag^+^ (1.0 Å) and Ga^3+^ (0.47 Å). This prevents cation intermixing or substitution, which is commonly observed in other chalcopyrite crystals like CuInS_2_, where the ionic radii are similar (0.74 Å for Cu^+^ and 0.76 Å for In^3+^) [13]. By employing a generalized gradient approximation method to calculate the electronic band structure of AgGaS_2_, Piasecki and colleagues found that the crystal displayed a nonzero direct energy gap at the Γ point (Figure 1c) [12]. The calculated bandgap (*E*_g_) using the GGA functional was determined to be 1.036 eV, slightly underestimating the experimental value of 2.65 eV.

The pioneering study of AgGaS_2_ bulk crystals can be traced back to the 1970s. The fundamental investigation of its NLO properties has stimulated intensive work on AgGaS_2_-related NLO crystals, especially in the infrared wavelength. In contrast, the development of AgGaS_2_ nanocrystals has faced significant challenges, primarily due to the complex wet-chemical synthesis required to obtain high-quality nanocrystals. Given the growing knowledge of reaction dynamics, the recent growth in research activity on wet chemistry has enabled the preparation of AgGaS_2_ nanocrystals with decent mono-dispersion and anticipated performance. The low toxicity, facile preparation, and flexible optical tunability of AgGaS_2_ nanocrystals make them promising for optoelectrical and lighting applications. In addition to the good optoelectrical properties, AgGaS_2_ has also inspired a series of derivative materials due to its intriguing NLO parameters. Therefore, the need for an in-depth study of AgGaS_2_ is self-evident, not only to provide an important scientific basis for future material optimization but also to help consolidate its position in existing applications.

Different from the currently existing reviews that mainly focus on the nonlinear properties of chalcogenides, we aim to provide a comprehensive overview of the current progress and future prospects in the design of AgGaS_2_ and its derivatives in this review. We focus on recent advancements in the understanding of their structural, electronic, and chemical properties. The review begins with an introduction to the synthesis of bulk AgGaS_2_ crystals in Section 2, which serves as the foundation for their utilization as NLO materials. Section 3 examines the NLO performance of AgGaS_2_ and its derivatives, presenting the guiding principles for the exploration of high-quality NLO materials. In Section 4, the chemical synthesis of AgGaS_2_ nanocrystals is discussed, highlighting the selection of appropriate reaction precursors and conditions. Finally, Section 5 summarizes the techniques employed to optically tune AgGaS_2_ nanocrystals through elemental alloying and core–shell engineering.

## 2. Bulk Crystal Synthesis

**Figure 2:**
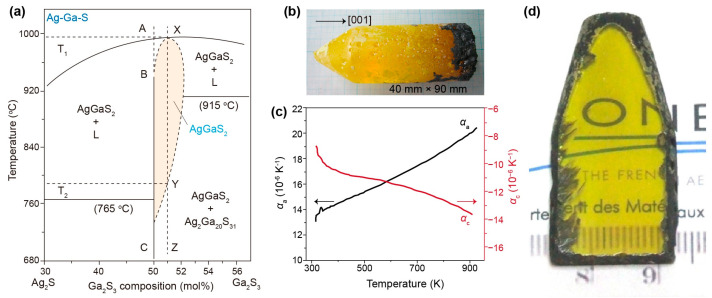
(**a**) Pseudo-binary phase diagram of the Ag_2_S–Ga_2_S_3_ system. Reproduced with permission [15]. Copyright 2007, Elsevier B.V. (**b**) Photograph of a AgGaS_2_ single crystal grown along the [001] direction with full size of 40 mm × 90 mm. (**c**) Temperature-dependent thermal expansion coefficients of a AgGaS_2_ crystal in the direction of the *a* and *c* axes, respectively. Reproduced with permission [18]. Copyright 2020, American Chemical Society. (**d**) Photograph of a AgGaS_2_ crystal annealed by alternant static and dynamic vacuum annealing. Note that the sample is 4 mm in thickness. Reprinted with permission [17]. Copyright 2010 Elsevier B.V.

AgGaS_2_ is a visually striking crystal with a transparent yellow color. It exhibits exceptionally high reflection optical activity, surpassing many other known materials [14]. With a melting point of ~996 °C (Figure 2a) [15], AgGaS_2_ single crystals are typically synthesized through melt growth using the vertical Bridgman or Czochralski method. From the pseudo-binary phase diagram, it is seen that AgGaS_2_ has good thermal stability at relatively high temperatures. It lies in a single-phase region at temperatures below 750 °C, above which it departs from the vertical line in the direction of Ga_2_S_3_. It is evidenced that single crystals grown from polycrystalline materials with near-stoichiometric compositions may contain excess Ga_2_S_3_. In an early study conducted by Route et al. in 1974, AgGaS_2_ single crystals with a diameter of 1.1 cm and length of 5 cm were successfully grown [16]. However, these crystals often suffered from issues such as the presence of precipitates, twinning, and even cracking, despite achieving optical clarity. The strong optical scattering caused by these imperfections hinders the practical application of low-quality crystals. Therefore, the primary challenge in the development of AgGaS_2_-based devices lies in the difficulty of growing sufficiently large crystals with high optical transparency [17,18].

### 2.1. Negative Thermal Expansion of AgGaS_2_ Crystals

Abnormal thermal expansion behavior during the crystal growth process is one of the main factors contributing to the difficulty of growing high-quality AgGaS_2_ crystals. It has been observed that AgGaS_2_ exhibits negative thermal expansion along the *c* axis when it cools from its melting point to room temperature, which often leads to the cracking of crystal ingots [19,20]. The exact thermal expansion coefficients *α*_a_ and *α*_c_ were recently determined by Huang and coworkers with a thermal dilatometer in the temperature range of 298–850 K [18]. The authors showed that *α*_c_ was −1.14 × 10^−5^ K^−1^ at 600 K, and the numerical value of *α*_a_ was larger than *α*_c_ in the whole temperature range (*α*_a_ > |*α*_c_|) (Figure 2b). This phenomenon can be attributed to the Ag/Ga vibrations of low-frequency transverse modes as well as the bond length change for *d*_Ag-S_ and *d*_Ga-S_ because the Rietveld refinement of in situ XRD patterns showed an increase in *d*_Ag-S_ and a decrease in *d*_Ga-S_ over the whole temperature range. As negative thermal expansion is an intrinsic nature of AgGaS_2_ crystals, slowing down the cooling steps along with the use of directed seed AgGaS_2_ may contribute to the success of growing high-quality AgGaS_2_ crystals. Current technology has allowed for AgGaS_2_ growth by using seed crystals oriented with the *c* axis parallel to the axis of the ampoule in which the crystal grows to avoid the cracking of the ampoule.

### 2.2. Second-Phase Precipitation of AgGaS_2_ Crystals

The other reason for the difficulty in growing high-quality AgGaS_2_ crystals is the occurrence of second-phase precipitation during cooling. According to the phase diagram, AgGaS_2_ crystallizes and grows in the two-phase region that is deviated from the stoichiometric line (Figure 2a) [15]. As a part of AgGaS_2_ will separate into Ga_2_S_3_ and Ag_2_S, AgGaS_2_ single crystals grown from a near-stoichiometric composition may contain excess Ga_2_S_3_ to form the intermediate phase Ag_2_Ga_20_S_31_ (Ga_2_S_3_ dissolving in solid AgGaS_2_), leading to the segregation of a thin, black Ag_2_S layer that deposits on the surface of AgGaS_2_ ingots (Figure 2c). The excessive precipitation of Ag_2_S further generates a fine-textured second phase containing Ga_2_S_3_ impurities due to supersaturation upon cooling at around 790 °C, which deteriorates the transparency [17]. Fortunately, the black Ag_2_S layer can be removed by mechanical polishing. Advances in crystal growth with suitable post-treatment have made large AgGaS_2_ single crystals with improved transparency available as well. For example, the inner Ga_3_S_2_ precipitates can be cleared by annealing at a temperature slightly below melting temperature in evacuated quartz chambers containing Ag_2_S. Moreover, the inner Ga_3_S_2_ precipitates can also be removed through a combination of static and dynamic vacuum annealing to balance sublimation and diffusion, as reported by Petit and coworkers [17]. Despite only a trace amount of Ga_2_S_3_ precipitates being removed during the dynamic stage (lasting for a few seconds) at 830 °C, the subsequent diffusion of excessive Ga and S species can slowly take place from the center of the bulk to the surface during the static stage (last for a few days). After several cycles, the concentration of Ga_2_S_3_ impurities could be decreased to an acceptable level. The successful preparation of large-size AgGaS_2_ crystals with few defects enables fundamental research on their NLO performance, and now AgGaS_2_ crystals are available on the commercial market [21,22].

## 3. AgGaS_2_ and Derivatives for Nonlinear Optics

NLO materials have been of great interest in recent decades because of their spectacular applications such as space communications, infrared countermeasures, medical diagnosis, and remote gas spectroscopy [23,24,25,26,27,28,29,30]. Governing SHG and phase-matching mechanisms are core concepts in modern NLO. The realization of phase matching is the key to ensure the efficiency of SHG, and its goal is to keep the phase of the incident light and its corresponding second harmonics consistent when propagating through the medium to reduce energy loss. Crystals belonging to the chalcopyrite symmetry class were proposed as possible infrared tunable optical parametric oscillation (OPO) sources as early as 1971 [31]. AgGaS_2_ crystals are transparent in the wavelength region of 0.45–13 µm with a nonlinear coefficient *d*_36_ of 11–13 pm/V and a birefringence Δn of 0.053 at 10.6 µm [32]. Note that such an adequate birefringence enables phase matching for both type-I (two low-frequency waves with the same polarization) and type-II (two low-frequency waves with orthogonal polarization) interactions, which are described by the following equations:

(4) $$for\;type\;I:\;d_{eff}=d_{36}sin\theta sin2\varphi$$

(5) $$for\;type\;II:\;d_{eff}=d_{36}sin2\theta sin2\varphi$$

where *d*_eff_ is the effective nonlinear coefficient, *d*_36_ is the nonlinear tensor component of AgGaS_2_, and *θ* and *φ* are the polar and azimuthal angles, respectively. Taking advantage of NLO properties, AgGaS_2_ crystals have been developed for frequency doubling, frequency mixing, and as parametric oscillators so that continuously tunable radiation from 1.0 to 12 µm can be produced under suitable pumping laser excitation. Notably, AgGaS_2_ is one of the few crystals that can be pumped by commercially available 1.06 µm lasers to achieve phase-matched downconversion into the λ > 5 µm region.

The groundbreaking utilization of AgGaS_2_ in OPO was first reported by Byer and colleagues, marking the first record of OPO in this class of chalcopyrite semiconductors [33]. The authors fabricated AgGaS_2_ crystals measuring 2 cm in length and 1.0 × 0.5 cm^2^ in cross-section, cut at an angle of 50.5° on the optical axis in the [110] direction. Pumped by an electro-optic *Q*-switched YAG: Nd laser (20 ns, 10 Hz), AgGaS_2_ OPO could achieve tunable emission in the wavelength range of 1.4 to 4 µm with type-I interactions. Although the operating wavelength of the AgGaS_2_ OPO could extend to the crystal’s transparency limit (~12 µm), the parametric oscillation threshold could not surpass λ > 4 µm due to significant damage occurring at the crystal surface. Subsequently, Vodopyanov and colleagues achieved the experimental realization of AgGaS_2_-based OPO with a wide mid-infrared tuning range [34]. The authors utilized AgGaS_2_ crystals measuring 2 cm in length and 0.7 × 1.0 cm^2^ in cross-sectional area. Narrow OPO linewidths were achieved, and effective second-order nonlinearity was maximized as well by choosing type-II phase matching, for which *θ*~45° yields *d*_eff_ = 12 pm/V, 25–30% higher than in the case of type-I (*θ*~52°). As a result, the singly resonant type-II AgGaS_2_ OPO pumped by a nanosecond YAG: Nd laser could yield an idler wave continuously tunable from 3.9 to 11.3 µm with a linewidth of 1 cm^−1^ and an OPO threshold of 0.03 J/cm^2^.

**Figure 3:**
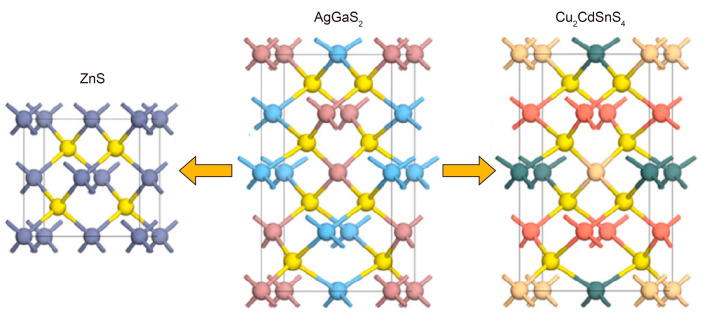
Structural evolution of diamond-like chalcogenides from II–VI (ZnS) to I–III–VI_2_ (AgGaS_2_), I_2_–II–IV–VI_4_ (Cu_2_CdSnS_4_). Reproduced with permission [41]. Copyright 2019, American Chemical Society.

Although AgGaS_2_ crystal possesses a wide transparency window and a large second harmonic generation (SHG) effect, its laser-induced damage threshold (LIDT) is rather small (that is, ~10 MW/cm^2^@1.06 µm) according to Vodopyanov’s report [34], owing to its relatively narrow bandgap (2.7 eV). Other commercially available infrared NLO materials such AgGaSe_2_ and ZnGeP_2_ also exhibit large SHG responses but still encounter the issue of low LIDT or two-photon absorption (TPA) [35,36]. The TPA effect of these materials will cause energy loss under a high power input, thus reducing the conversion efficiency. Moreover, Ag-containing NLO materials like AgGaS_2_ additionally suffer from the photodarkening effect of elemental Ag, which dramatically shortens the crystal longevity. These drawbacks to some extent hinder their practical applications for long-term use. In this regard, it is desirable to explore novel alternative NLO materials that exhibit better NLO performance in the infrared spectral region [37,38,39,40]. In the literature, it is well recognized that the optical properties of an ideal infrared NLO material should meet the following criteria: (i) the infrared transparency window should be broad enough to at least cover two important atmospheric transparent windows (i.e., 3–5 µm and 8–12 µm); (ii) the SHG coefficient *d*_ij_ should be large, at least larger than 10 × KDP (KH_2_PO_4_, with *d*_36_ = 0.39 pm/V); (iii) the LIDT should be higher than that of AgGaS_2_, and the corresponding bandgap should be larger than ~3.0 eV; (iv) the birefringence Δn should fall in a suitable range (i.e., 0.03–0.10) to achieve the NLO phase-matching condition; and (v) the crystal should have a good growth habit and chemical stability. Taking chalcopyrite AgGaS_2_ as a model, the alternatives are mainly focused on its derivatives possessing a similar diamond-like structure (Figure 3) [41].

In a classic diamond-like ZnS material, the Zn–S bond lengths within the [ZnS_4_] group have a narrow range of 2.339–2.343 Å, with only a slight difference of 0.004 Å. The S–Zn–S angles are located in the range of 109.65–109.68°, which has only a very small deviation from the standard tetrahedral structure (109.5°). As a result, binary diamond-like materials such as ZnS are not suitable for infrared SHG applications due to their limited structural anisotropy, resulting in small birefringence [37]. As an alternative, diamond-like ternary or quaternary compounds derived from AgGaS_2_ with suitable phase-matching properties are more promising [42,43]. These compounds exhibit relatively weak refractive index dispersion in the long wavelength region, allowing for a relatively small birefringence (Δn ~ 0.03–0.07) to satisfy the phase-matching condition in the UV-to-infrared spectral range. Excessive birefringence (Δn > 0.10) can lead to destructive optical frequency conversion due to large walk-off and self-focus effects, which impede the generation of high-power coherent light [44,45]. Various sulfide units, such as [PS_4_], [SiS_4_], [GeS_4_], [InS_4_], [CdS_4_], [ZnS_4_], and [LiS_4_] can be substituted for the fundamental tetrahedral building blocks [AgS_4_] and [GaS_4_] in the diamond-like 3D framework. These tetrahedra can be interconnected and aligned, enabling the additive superposition of the microscopic second-order susceptibility tensors. It is worth noting that finding an ideal NLO material with a wide energy gap (*E*_g_ > 3.0 eV) and a large SHG efficiency (greater than that of AgGaS_2_) is highly desirable but remains a significant challenge due to the inherent trade-off between the SHG coefficient and bandgap value. Increasing the bandgap excessively will lead to a decrease in the SHG coefficient. To this end, the balanced SHG coefficient and bandgap value of AgGaS_2_ provide it with good application prospects. In the pursuit of efficient and reliable NLO materials, AgGaS_2_ is obviously an important reference. Further advancements in performance and quality focus on modifying the bandgaps or SHG coefficients, as summarized in Table 1.

### 3.1. Bandgap Modification

**Figure 4:**
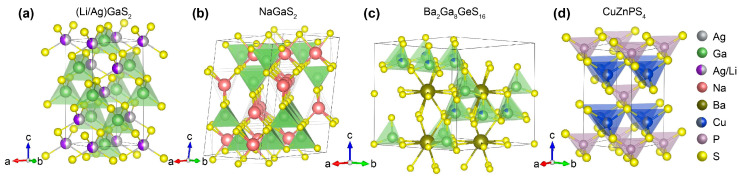
Crystal structures of (**a**) (Li/Ag)GaS_2_, (**b**) NaGaS_2_, (**c**) Ba_2_Ga_8_Ge_16_, and (**d**) CuZnPS_4_ showing mixed-anion polyhedrons arranged in diamond-like configurations.

The narrow bandgap of AgGaS_2_ is attributed to the presence of the 3d orbitals of Ag at the valence band maximum [37,58,59,60,61]. To modify the bandgap and improve the LIDT, the incorporation of an electropositive element, such as an alkaline metal (e.g., Li and Na) or alkaline earth metal (e.g., Ba), into the Ag sites is considered. These elements do not exhibit d-d or f-f electron transitions [62]. For instance, replacing [AgS_4_] with [LiS_4_] results in the formation of LiGaS_2_, which possesses a significantly larger bandgap of 3.7 eV and exhibits a notably higher LIDT (11 times that of AgGaS_2_) [51]. However, when Li^+^ is substituted for Ag^+^ in AgGaS_2_, the space group of AgGaS_2_ collapses into a lower *P*na2_1_ orthorhombic symmetry. As a result, the NLO coefficient of LiGaS_2_ is significantly reduced (*d*_36_ = 5.8 pm/V), and its SHG performance is inferior to that of AgGaS_2_. Considering that Li^+^ and Ag^+^ share many chemical similarities, such as the same valence state and four-fold tetrahedral coordination in a compound, some researchers have suggested that Li^+^ might be dissolved in AgGaS_2_ to form a solid solution, while the space symmetry could be preserved. This idea was proposed by Wu and coworkers, who examined a series of Li_x_Ag_1-x_GaS_2_ crystals in 2019 [53]. The authors found that for Li_x_Ag_1-x_GaS_2_, Li^+^ substitution did not change the crystal structure when *x* was tuned from 0 to 0.6. Correspondingly, the bandgaps can be fine-tuned from 2.58 (x = 0, AgGaS_2_) to 3.40 eV (x = 0.6, Li_0.6_Ag_0.4_GaS_2_) (Figure 4a). The large bandgap of Li_0.6_Ag_0.4_GaS_2_ enables an 8.6-fold enhancement in the LIDT at 1.06 µm compared to that of AgGaS_2_. This improvement may be attributed to the reduced difference in cationic radii between Ag^+^ (1.0 Å) and Ga^3+^ (0.47 Å) through Li^+^ (0.59 Å) substitution. In mixed-cation crystals like Li_0.6_Ag_0.4_GaS_2_, dislocations can be modified, shifting the structure back toward the ideal diamond-type structure and allowing for better alignment of the [GaS_4_] units along the chalcopyrite direction.

In addition to the Li^+^ substitution, Na^+^ or Ba^2+^ substitution is also feasible. It should be noted that the introduction of the Na^+^ cation will break the 3D [GaS_4_] framework into 2D layers [63,64]. In NaGaS_2_ crystals, Ga atoms are four-coordinated with S atoms to form [GaS_4_] tetrahedra, and every four tetrahedra gather to form a super-tetrahedral [Ga_4_S_10_] cluster through corner sharing (Figure 4b). The unique packing configuration results in a large bandgap of 3.9 eV and a high birefringence of ~0.09 [48]. Meanwhile, the blue shift of the UV cutoff edge to 307 nm is observed, suggesting promise as a functional material working in the visual-to-near-IR region. However, the authors have not characterized the SHG coefficient *d*_ij_ of the NaGaS_2_ crystals. By inspecting the symmetry of NaGaS_2_, there are reasons to believe that the SHG performance cannot surpass that of AgGaS_2_. The introduction of alkaline earth elements such as Ba into the diamond-like sulfide framework can also modify the bandgap [65]. For example, Dong and coworkers found that Ba_2_Ga_8_SiS_16_ and Ba_2_Ga_8_GeS_16_ adopted a 3D framework structure exhibiting the alternate stacking of two distinct tetrahedral layers (Figure 4c) [66]. The samples adopted the same *P*6_3_*mc* space group and had large bandgaps (3.4 eV for Ba_2_Ga_8_SiS_16_ and 3.0 eV for Ba_2_Ga_8_GeS_16_) and a broad transparency window (0.42–20 µm). Particularly, Ba_2_Ga_8_GeS_16_ exhibited an LIDT ~22 times higher than that of AgGaS_2_ owing to the large bandgap. Ba_2_Ga_8_GeS_16_ also exhibited strong SHG signals that were comparable to those of the benchmark AgGaS_2_ at a laser irradiation of 1.95 µm. The strong nonlinear effect was presumably related to the synergic effect of the alternate stacking of the mixed [(Ga/Ge)S_4_] and the pure [GaS_4_] tetrahedral layers along the *c* axis, the alignment of these two types of tetrahedra in the same direction, and the slightly off-center feature of alkaline earth cations in the interstitial sites.

Apart from alkaline and alkaline earth substitution, the introduction of P^5+^ into chalcogenide polyhedra has also been reported to enlarge bandgaps [67]. Metal thiophosphate can intrinsically satisfy the crucial criteria for a promising infrared NLO crystal due to the strong covalent P–S bonds [68,69]. Moreover, thiophosphate exhibits diverse P–S anionic groups such as [PS_4_] and [P_2_S_6_] [70], which can further connect with other NLO-active units to construct varied crystal architectures, giving rise to more possibilities for designing NLO crystals. It should be mentioned that thiophosphates usually possess a relatively low melting point, and this feature certainly favors large crystal growth. Guided by this idea, Yao and coworkers designed a novel thiophosphate CuZnPS_4_ that showed a sharply enlarged energy gap (3.0 eV) and reinforced the SHG response (*d*_14_ = 15.9 pm/V) (Figure 4d) [67]. The lengths of the Cu/Zn–S and P–S bonds were 2.3227 and 2.0426 Å, which were shorter than those of Ag–S (2.605 Å) and Ga–S (2.235 Å) bonds in AgGaS_2_, implying stronger covalent interactions in the CuZnPS_4_. With the assistance of density functional theory calculation, the authors indicated that the highest occupied atomic orbitals (HOMO) of CuZnPS_4_ were dominated by earring-shaped S 3p electrons, while the lowest unoccupied atomic orbitals (LUMO) primarily comprised S 3p and P 3p electrons. Owing to the occurrence of nonbonding states, the electron density in the adjacency of the forbidden gap of CuZnPS_4_ was significantly increased in comparison to that of AgGaS_2_.

The introduction of an alkaline metal (Li^+^ and Na^+^), alkaline earth metal (Ba^2+^), or P^5+^ modifies the bandgap by changing the crystal structure and the local electronic environment. However, the introduction of additional elements may lead to the distortion of the crystal structure, especially at high concentrations. This leads to a decline in optical properties and a weakening of material stability under fluctuant environmental factors such as humidity and temperature.

### 3.2. SHG Modification

**Figure 5:**
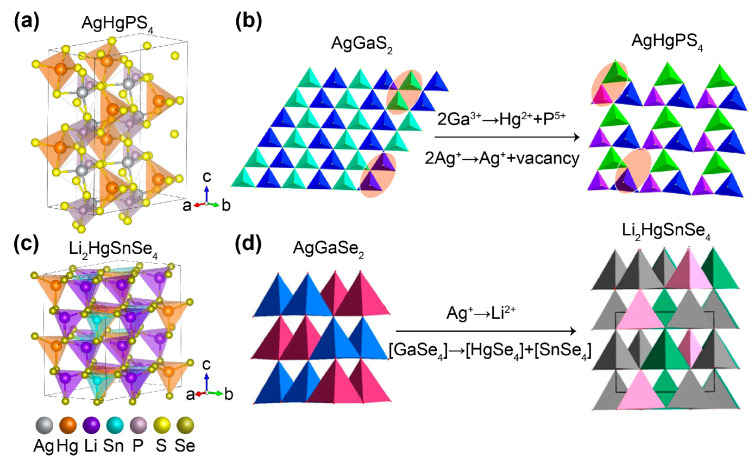
(**a**) Crystal structure of AgHgPS_4_. (**b**) Structural evolution from AgGaS_2_ to AgHgPS_4_. Reproduced with permission [56]. Copyright 2021, The Royal Society of Chemistry. (**c**) Crystal structure of Li_2_HgSnSe_4_. (**d**) Structural evolution from AgGaSe_2_ to Li_2_HgSnSe_4_. Reproduced with permission [57]. Copyright 2022, American Chemical Society.

The SHG response is largely related to the coordination and polarization of the polyhedron. Compounds containing transition-metal cations (e.g., Cu^+^, Zn^2+^, Cd^2+^, and Hg^2+^) with an (n-1)d^10^ns^0^ configuration or main-group metal cations (e.g., Pb^2+^, Sn^2+^, Bi^3+^, and Te^4+^) with an ns^2^ configuration are potential candidates for polar crystals. It is known that the former elements have a polar displacement of d^10^ cation centers, while the latter ones undergo the second-order Jahn–Teller (SOJT) effect caused by lone-pair electrons [71]. Among these tetrahedra, Hg^2+^-containing diamond-like sulfides have been widely studied because Hg is the heaviest member in the d^10^ group, and Hg–S bonds are highly polarizable, which is believed to significantly increase the SHG response and birefringence. In addition, [HgS_4_] tetrahedra exhibit a larger distortion compared to other tetrahedra like [ZnS_4_] and [CdS_4_] [72]. As a typical example, Yao and coworkers reported the replacement of [GaS_4_] units by highly distorted [HgS_4_] and [PS_4_] units for the preparation of the new NLO material AgHgPS_4_ with a bandgap of 2.63 eV (Figure 5a,b) [56]. The authors calculated the contributions of [AgS_4_] (−10.64 pm/V), [HgS_4_] (−30.77 pm/V), and [PS_4_] (−11.54 pm/V) units and concluded that the [HgS_4_] units contributed most to the phase-matching SHG response (*d*_11_ = −31.08 pm/V). Moreover, they demonstrated that this material system could be further extended to a uniform structure of AHgPS_4_ (A = Li and Cu), demonstrating the great advantages of the combination of cation vacancy defects and highly distorted [HgS_4_] tetrahedra. In a parallel contribution, Wu and coworkers reported designed and synthesized Li_2_HgMSe_4_ (M = Si, Ge, and Sn) NLO materials by concurrently replacing the cation Ag^+^ and the [GaSe_4_] unit with the alkali metal Li^+^ and [HgSe_4_]/[MSe_4_] to optimize crystal structures and performances (Figure 5c,d) [57]. Optical characterization showed that Li_2_HgMSe_4_ exhibited bandgaps of 1.2–1.7 eV and strong powder SHG responses (3.6–6.0 × AgGaS_2_) with the essential phase-matching behavior (Δn = 0.042 − 0.172). Particularly, Li_2_HgSnSe_4_ exhibited the largest SHG response (*d*_33_ = −104.3 pm/V) among the known Hg-based chalcogenides. This research highlights the practicability of the functional group co-substitution strategy and shows that Hg-based chalcogenides can be a promising system for the future exploration of large SHG crystals.

It is known that the distribution of valence electrons and the spatial arrangement of active groups are two determinants of NLO response. Although the [AgS_4_] and [GaS_4_] tetrahedra in AgGaS_2_ already occur in a favorable way with co-parallel alignment and high density, there is still room for improving NLO performance by fine-tuning the valence electron distribution through doping or by designing tetrahedral positions through multiple doping.

According to the valence electron concentration (VEC) theory, the VEC can be defined using the following equation [67]:

(6) $$VEC=\frac{me_{A}+ne_{B}+\cdots }{m+n+\cdots }$$

where *e*_A_ and *e*_B_ are the valence electron numbers of compositional elements A and B in the [AB_4_] tetrahedral unit; *m* and *n* represent the atomic numbers of A and B, respectively. Guided by this principle, a normal diamond-like structure such as that of AgGaS_2_ adopts a VEC number of 4. With the introduction of an element with high electrovalence such as Ge^4+^ in a normal diamond-like compound, the VEC number will obviously increase owing to the occurrence of cation vacancies. For example, Hg_2_GeSe_4_ shows a large VEC number of 4.57 [73]. By first-principle calculation, Lin and coworkers found that Hg_2_GeSe_4_ has a bandgap of 2.8 eV, a moderate birefringence value of 0.096, and a large SHG coefficient of *d*_36_ = 38.22 pm/V [71].

**Figure 6:**
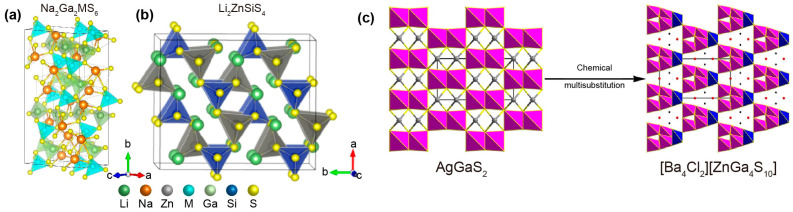
(**a**) Crystal structure of Na_2_Ga_2_MS_6_ (M = Ge or Sn). (**b**) Crystal structure of Li_2_ZnSiS_4_. Reproduced with permission [76]. Copyright 2022, American Chemical Society. (**c**) Crystal structure of [Ba_4_Cl_2_][ZnGa_4_S_10_]. Reproduced with permission [50]. Copyright 2020, American Chemical Society.

The idea of the spatial arrangement of active groups can be realized by combining two or more different typical NLO-active motifs, such as [GaS_4_], [BaS_4_], [InS_4_], [GeS_4_], and [SiS_4_] tetrahedra, forming super-polyhedral clusters in a single chalcogenide compound to modulate SHG efficiency and LIDT simultaneously [52,74,75]. One can imagine that multiple polyhedra in the structure will lead to large SHG responses. Guo and coworkers presented two infrared NLO materials, Na_2_Ga_2_GeS_6_ and Na_2_Ga_2_SnS_6_, by mixing the different typical NLO-active motifs [GaS_4_] and [GeS_4_]/[SnS_4_] into the alkali metal-containing system (Figure 6a) [55]. Optical characterization showed that Na_2_Ga_2_GeS_6_ and Na_2_Ga_2_SnS_6_ had optical bandgaps of 3.1 and 2.74 eV and *d*_eff_ values of 11.18 and 13.11 pm/V. In another work guided by the strategy of co-substitution, Zhou and coworkers reported the preparation and optical characterization of Li_2_ZnSiS_4_ crystals [49]. The structure of Li_2_ZnSiS_4_ can be considered a replacement of the Ag and [GaS_4_] units by alkali metal Li [ZnS_4_]/[SiS_4_] units (Figure 6b) [76]. Theoretical calculations and optical characterizations indicated that Li_2_ZnSiS_4_ showed a large bandgap of 3.9 eV, a broad transparency window range of 3–25 µm, a high LIDT (10 × AGS), and a large phase-matching SHG response of *d*_33_ = 18.89 pm/V at 2.09 μm. This co-substitution idea was further extended to the salt-inclusion chalcogenides [R_a_X_b_][MS_4_] (R = alkaline or alkaline earth elements; X = halide elements; and M = main-group or/and transition elements), where tetrahedral [MS_4_] works as an asymmetric building unit, and ionic guest [R_a_X_b_] provides filler ions to stabilize the structures [77,78,79]. For example, Wu and coworkers designed the salt-inclusion chalcogenide [Ba_4_Cl_2_][ZnGa_4_S_10_] in accordance with the AgGaS_2_ template (Figure 6c) [50]. Structural characterization showed that [Ba_4_Cl_2_][ZnGa_4_S_10_] was constructed from corner-sharing super-tetrahedral [Ga_4_S_10_] clusters and [ZnS_4_] tetrahedra to form a three-dimensional open [ZnGa_4_S_10_] diamond-like framework. Ba^2+^ and Cl^−^ ions interpenetrated the framework to support charge balance. Further optical characterization indicated that the [Ba_4_Cl_2_][ZnGa_4_S_10_] crystal possessed a wide bandgap of ~3.85 eV, a large SHG coefficient of *d*_14_ = 14.9 pm/V, and an ultrahigh LIDT of 51 × AGS, demonstrating its promise as an infrared NLO candidate.

It should be emphasized that all diamond-like ternary or quaternary materials are SHG active because of their inherently non-centrosymmetric structures. However, when the overall performances are compared, it seems that AgGaS_2_ is still one of the most useful NLO materials since it possesses balanced NLO properties [40,80]. Therefore, many studies are still focused on the classic AgGaS_2_. For example, Cheatum and coworkers presented a method for increasing the power of mid-infrared laser pulses generated by AgGaS_2_ difference frequency generation, amplifying mid-infrared light twofold [28]. Indeed, most of the other known ternary and quaternary diamond-like structure materials exhibit either smaller bandgaps or weaker SHG effects [81]. One particular material, that is, Li_2_ZnSiS_4_, needs further systematic study since it presents a superior balance between the bandgap (>3.0 eV) and the SHG effect (>AgGaS_2_), as mentioned in the above discussion [41]. This result implies that although it seems challenging to find an optimized infrared NLO material, it is possible to design a target material by using AgGaS_2_ as a model for quaternary diamond-like metal sulfides.

## 4. Nanocrystal Synthesis

The traditional solid-state reaction for synthesizing AgGaS_2_ involves high processing temperatures (800–1000 °C), long reaction times, strict sintering processes, and specialized equipment. In contrast, the wet-chemical synthesis of AgGaS_2_ nanocrystals is more straightforward and time-saving. By reducing the crystal size to the excitonic Bohr radius (3.3 nm for AgGaS_2_) [82,83,84,85,86], the restricted electron movement results in wideband light absorption and bright light emission, known as the quantum confinement effect [87,88]. Moreover, the large surface-to-volume ratio of nanocrystals allows for flexible surface modification, leading to extended functionalities. While there has been extensive research on chalcopyrite semiconductor nanocrystals such as CuInS_2_ [89], CuGaS_2_ [90], and CuInSe_2_ [91], AgGaS_2_ nanocrystals are not as well explored, mainly due to the challenges associated with incorporating Ag.

### 4.1. Solvothermal Synthesis

Early attempts to synthesize nanocrystalline AgGaS_2_ were made by Qian and coworkers in the late 1990s [92]. Through a standard solvothermal synthetic route, the authors prepared tetragonal AgGaS_2_ nanocrystals 5–7 nm in diameter by using AgCl, Ga, and S as the reactants at 180–230 °C. In a modified reaction, they used AgCl, GaCl_3_, and thiourea as reactants [93]. Interestingly, the introduction of thiourea interacted with AgCl to form a water-soluble Ag–thiourea complex in a homogenous solution, which was helpful to form nanocrystalline AgGaS_2_ (~5 nm in diameter). Consequently, spectral results showed that the photoluminescence spectrum consisted of one broad emission feature at 446 nm, which was blue shifted at about 50 nm compared with that of single-crystal AgGaS_2_, indicating the quantum confinement effect. However, the AgGaS_2_ nanocrystals prepared via the hydrothermal route at that time were inclined to form assembled microparticles, attributed to the lack of proper surface ligands. Through a solvothermal synthesis, AgGaS_2_-based nanohybrids can be easily formed. In a recent study, Wang and colleagues found that the attachment of AgGaS_2_ nanosheets to the edges of carbon nitride resulted in the formation of a type-II heterostructure, exhibiting an enhanced photocatalytic property [85]. The following contributions mainly focus on the synthesis of monodispersed AgGaS_2_ nanocrystals though bench-top methods employing heat-up and hot-injection techniques (Table 2).

### 4.2. Heat-Up Synthesis

The successful growth of AgGaS_2_ nanocrystals through a heat-up procedure typically involves fast nucleation and subsequent crystal growth, as described by the classic LaMer model [106]. Compared to the classic binary quantum dots, the formation of uniform AgGaS_2_ nanocrystals in the Ag–Ga–S ternary system is more complicated because the reactivity of double cations should be balanced in addition to temporally separating the nucleation and growth processes. To restrain dual nucleation processes, the reaction activities of two metal precursors should not differ significantly from each other, which is identified as the key to deliberately tuning the electronic band feature of the nanocrystals.

**Figure 7:**
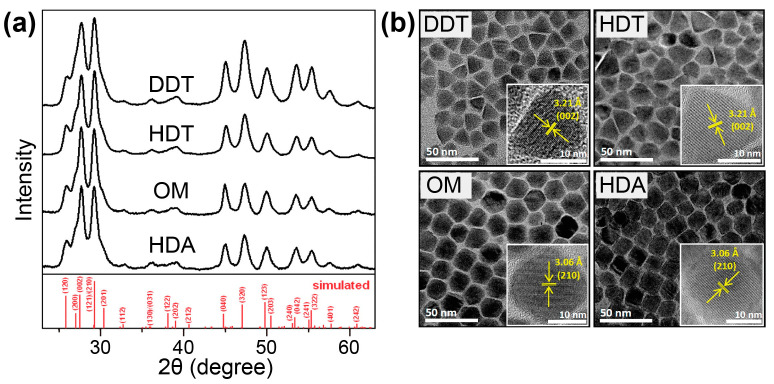
(**a**) XRD pattern and (**b**) TEM images of AgGaS_2_ nanocrystals prepared using dodecanethiol (DDT), hexadecanethiol (HDT), oleylamine (OM), and hexadecylamine (HAD). Reproduced with permission [94]. Copyright 2014, The Royal Society of Chemistry.

A previous study reported that when single-source AgGa(S_2_CN(C_2_H_5_)_2_)_4_ was used as the precursor and oleylamine was used as the solvent, AgGaS_2_ nanocrystals could be obtained from a reaction at 180–240 °C for 10 min [97]. Later, Han and coworkers reported the synthesis of AgGaS_2_ nanocrystals using AgS_2_CN(C_2_H_5_)_2_ and Ga(S_2_CN(C_2_H_5_)_2_)_3_ complexes separately in conjunction with proper coordination solvents [94]. Structural characterization showed that pure-phase AgGaS_2_ nanocrystals were obtained when long-chain alkanethiols (dodecanethiol and hexadecanethiol) or alkylamines (oleylamine and hexadecylamine) were employed (Figure 7). It should be noted that in this case, the final products were in an orthorhombic phase rather than a common tetragonal phase. When oleic acid was employed as solvent, AgGaS_2_ nanocrystals were not successfully prepared, and instead, only a mixture of Ag and Ag_2_S was produced; when a tertiary alkylamine such as trioctylamine was utilized, a mixture of tetragonal AgGaS_2_ and Ag_9_GaS_6_ was produced; and if trioctylphosphine was used, only Ag crystals were yielded without any Ga-associated byproducts. Moreover, although the preparation of AgGaS_2_ nanocrystals was feasible using this method, the crystal sizes were excessively large (>15 nm); therefore, they were beyond the scope of the quantum confinement, and thus little photoluminescence was recorded.

In addition to the synthesis of AgGaS_2_ nanocrystals with a hydrophobic nature, the direct synthesis of nanocrystals with a hydrophilic surface is favored for biomedicine-related applications. This idea was realized recently in a demonstration reported by Azhniuk and coworkers [96]. The colloidal synthesis of AgGaS_2_ nanocrystals was carried out in a coprecipitation reaction between Na_2_S and a mixture of Ag(I) and Ga(III) thiolate complexes in aqueous glutathione solutions at 93–98 °C. Glutathione acted as not only the S source but also as the coordinating ligand so that small AgGaS_2_ nanocrystals with a size of ~2 nm could be obtained, and a blue shift of the photoluminescence peak was observed in comparison to that of the reported large nanocrystals.

### 4.3. Cation-Exchange Synthesis

**Figure 8:**
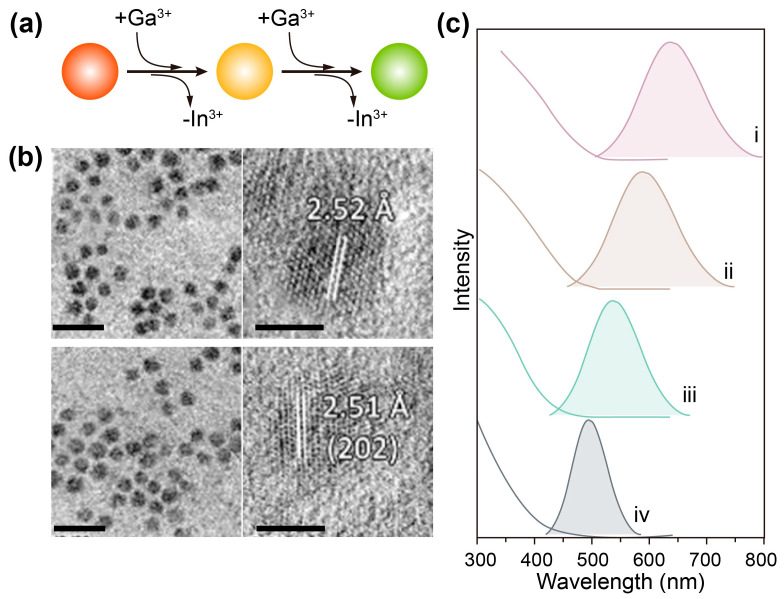
(**a**) Scheme of the synthesis of AgGaS_2_ nanocrystals through a cation-exchange approach. (**b**) TEM images and high-resolution TEM images of AgGa_x_In_1-X_S_2_ nanocrystals (top panel) and AgGaS_2_ nanocrystals (bottom), respectively. Scale bars are 10 nm and 3 nm for TEM images and high-resolution TEM images. (**c**) Absorption and emission spectra of nanocrystals with [Ca]/[In] ratios of (i) 0.85, (ii) 1.56, (iii) 2.88, and (iv) 3.73 in precursors, respectively. Reproduced with permission [98]. Copyright 2019, American Chemical Society.

To control crystal size, synthesizing AgGaS_2_ nanocrystals through a cation-exchange method was explored by Hughes and coworkers [95]. The formation of AgGaS_2_ nanocrystals was initiated by the rapid injection of elemental sulfur in dodecanethiol into a reaction vessel containing AgNO_3_ and Ga(acac)_3_ in octadecene at 170 °C. The authors observed an immediate change in solvent color from colorless to black and finally to translucent red–brown, indicating the formation of Ag_2_S at the early stage. This result also implies a potential cation exchange from Ag_2_S to AgGaS_2_ during the synthesis, which was proven by the sample characterization of aliquots taken at very early times. A deliberate cation-exchange synthesis method for AgGaS_2_ was developed by Song and coworkers, who used the as-synthesized AgInS_2_ nanocrystals as templates (Figure 8) [98]. As a thermal dynamic and kinetic process, nanoscale cation-exchange reactions generally consist of four steps driven by solvation energy and Lewis acid–base interactions: (i) extraction of the surface host cations; (ii) incorporation of guest cations into the nanocrystal surface; (iii) diffusion of the surface guest cations into the interior of nanocrystals; and (iv) diffusion of the inner host cations to the nanocrystals surface. In this regard, the cation exchange rate is in a close relationship with the cation concentration. In the demonstration by Song’s team, at an initial ratio of [Ga]/[In] = 3.73, the In^3+^ ions were entirely replaced by Ga^3+^ ions, leading to the formation of AgGaS_2_ with a stoichiometry of [Ag]/[Ga]/[S] = 1: 0.98: 2.11. Owing to the small ionic radius of Ga^3+^ (0.47 Å) in comparison with that of Ag^+^ (1.0 Å) and the strong bond strength of Ag–S, the Ag framework was well preserved during the cation-exchange process. The photoluminescence peak of the resulting nanocrystals could be tuned from 502 to 719 nm by regulating the reaction conditions, with the highest quantum yield of up to 37%.

We understand that for small nanocrystals like quantum dots, the crystal sizes and morphologies play a great role in their optical properties, attributing to the quantum confinement effect. Indeed, the preparation of AgGaS_2_ nanocrystals, especially the role of surface ligands in stabilizing AgGaS_2_ nanocrystals, is still an area that requires further exploration and research. Small changes in the reaction conditions (such as pH, temperature, reaction time, etc.) in wet chemistry may affect the properties of the product, which requires strict process control and optimization. Currently, there are limited data available on the synthesis of AgGaS_2_ nanocrystals, whether through direct synthesis or cation-exchange methods. Due to the potential use of AgGaS_2_ nanocrystals as blue lighting sources, there is a high demand for the development of wet-chemical synthesis methods. To advance the field, it is important to gain a deeper understanding of the reaction dynamics involved in the formation of AgGaS_2_ nanocrystals. This can be achieved through in-depth examinations of nanocrystal formation processes, as well as investigations into the composition of the nanocrystals using in situ microscopy techniques. By studying the reaction dynamics and nanocrystal composition, researchers can gain valuable insights into the factors influencing the synthesis process and make improvements to achieve the desired nanocrystal properties. It should be mentioned that with the aid of theoretical and simulation methods and tools, researchers can now guide the synthesis of novel semiconductors using a predictable approach. For example, Filho and coworkers revealed the self-induced formation and stability of InAlN nanorods through ab initio simulations and a density functional theory-based phase-field model [107,108]. It is certainly possible that such a method can be applied to guide the synthesis of AgGaS_2_ nanocrystals.

## 5. Optical Tuning of AgGaS_2_ Nanocrystals for Lighting and Photovoltaic Applications

The limited optical tunability of pristine AgGaS_2_ nanocrystals can be overcome by employing strategies such as element alloying and core–shell configurations. These approaches allow for precise control of the nanocrystal composition, enabling energy-level engineering and fine-tuning of the emission properties. By introducing alloying elements into AgGaS_2_ nanocrystals, the energy levels and bandgaps can be modified, leading to tunable emission properties. This alloying process involves incorporating different elements into the crystal lattice, which alter the electronic structure and introduce new energy states. In addition to alloying, the development of core–shell structures in AgGaS_2_ nanocrystals provides another avenue for emission tuning. The shell can modify the electronic and optical properties of the nanocrystals, leading to wavelength and intensity tuning of the emitted light. By varying the composition and thickness of the shell layer, precise control over the emission properties can be achieved. By leveraging these strategies, it is now possible to engineer energy levels and fine-tune emission properties, expanding the range of potential applications for AgGaS_2_ nanocrystals.

### 5.1. Element Alloying for Lighting Applications

**Figure 9:**
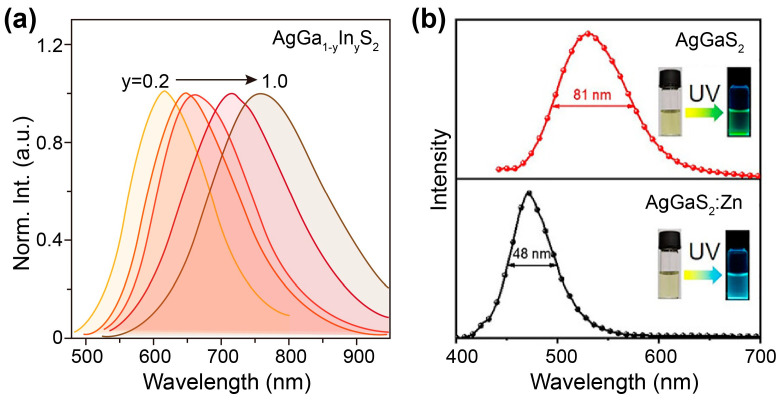
(**a**) Normalized photoluminescence spectra (λ_ex_ = 470 nm) of AgGa_1−y_In_y_S_2_ (y = 0.2~1.0) nanocrystals. Reproduced with permission [97]. Copyright 2010, American Chemical Society. (**b**) Normalized photoluminescence spectra of AgGaS_2_ and AgGaS_2_: Zn nanocrystals. Insets: corresponding photos of AgGaS_2_ and AgGaS_2_: Zn nanocrystals under natural light and 365 nm ultraviolet light. Reproduced with permission [83]. Copyright 2022, American Chemical Society.

Element alloying has been widely adopted to tune the bandgap energy and photoluminescence of semiconducting quantum dots through band mixing, according to Vegard’s law [109]. For AgGaS_2_, Ga^3+^, and S^2−^, sites can be alloyed by In^3+^ and Se^2−^ ions, which greatly tune their absorption and emission bands. Given the structural similarity of AgGaS_2_ and AgInS_2_, Ga^3+^-site engineering was reported early in 2010 [97]. Kuwabata and coworkers reported the synthesis and emission of AgIn_x_Ga_1−x_S_2_ (0 < *x* < 1) nanocrystals via the thermal decomposition of a metal ion–diethyldithiocarbamate complex in oleylamine. Inductively coupled plasma atomic emission spectroscopy (ICP-AES) results confirmed that the ratios of [Ga] to [In] in the resulting nanocrystals were almost equal to those in the precursors, implying the complete thermal decomposition of the precursors. Spectroscopy results showed that the increase in the [Ga]/[In] ratio led to a red shift from 550 to 750 nm in the photoluminescence spectra, attributed to the band mixing of AgInS_2_ (~1.8 eV) and AgGaS_2_ (~2.6 eV) (Figure 9a). Notably, the alloyed nanocrystals showed high quantum yields of ~40%, although the pristine AgGaS_2_ nanocrystals were almost non-luminescent. It should be mentioned that due to the different reaction activities between Ga and In precursors (usually, Ga precursors have a low activity because of their small ionic radius), the cation distribution may not be uniform in the single nanocrystals. For example, it has been observed that AgInS_2_ nanocrystals could be synthesized at 150 °C, while AgGaS_2_ nanocrystals formed only at a temperature over 250 °C under the same reaction conditions. In this regard, the balance of cation reactivity is essential in the synthesis of real AgIn_x_Ga_1−x_S_2_ nanocrystals. Recently, researchers developed a synthetic route using multiple precursors to ensure balance [104]. They used gallium diethyldithiocarbamate (Ga(DDTC)_3_) as the target precursor, which reacts under moderate conditions to supply both Ga and S. The use of this precursor with silver acetate and indium acetate allowed for the synthesis of AgIn_x_Ga_1−x_S_2_ nanocrystals at 150 °C.

In addition to In^3+^ alloying, Zn^2+^ alloying is also possible, as the ionic diameter of tetragonally coordinated Zn^2+^ (0.60 Å) is comparable to that of Ga^+^ (0.47 Å), which thus can be preferentially substituted by Zn^2+^. For example, Yang and coworkers found that when Zn was alloyed, the emission of AgGaS_2_: Zn nanocrystals could be tuned from 515 to 450 nm [100]. In another contribution, Tang and coworkers reported the synthesis of narrow-bandwidth blue-emitting AgGaS_2_: Zn nanocrystals via a facile one-pot method [83]. When the Ag/Zn and Ag/Ga feeding ratios were selected with values of 4:1 and 1:8, respectively, AgGaS_2_: Zn nanocrystals demonstrated a typical blue emission at 470 nm with narrow full width at half-maximum (FWHM) of 48 nm and a high PLQY of 16.7% (Figure 9b), which was suitable for solution-processed quantum-dot light-emitting diode (LED) applications. It should be noted that due to the difference in chemical reactivities between Ga and Zn, the chemical composition of AgGaS_2_: Zn nanocrystals is probably still nonuniform. In fact, in one-pot synthesis, Zn is potentially concentrated in the interior of the AgGaS_2_: Zn rather than forming a shell, since the reactivity of the Zn precursor is a little higher than that of the Ga precursor, which has been evidenced by energy-dispersive X-ray spectroscopy. Despite this inhomogeneity, the role of Zn is still prominent in promoting emission efficiency. Tang and coworkers attributed the emission promotion to the increase in the emission center, induced by this hetero-valent substitution. The addition of an appropriate Zn precursor for one-pot synthesis can produce more Ag^+^ vacancies, which are responsible for charge carrier recombination. In addition, of course, the introduction of the appropriate amount of Zn can form a layer of ZnS, providing surface passivation for luminescence quenchers.

Apart from the above example, S^2−^-site alloying with Se^2−^ to form AgGa(S_1–x_Se_x_)_2_ is also feasible, and such alloying leads to a red shift in both the absorption and emission spectra [99]. Furthermore, multiple sites can be alloyed simultaneously, thereby greatly expanding the absorption and emission properties [110]. However, due to the unbalanced reactivity, multiple alloying requires more precise control over the uniform growth of alloyed nanocrystals during the entire synthesis procedure, which is promising but yet to be developed.

### 5.2. Core–Shell Engineering for Lighting and Photovoltaic Applications

**Figure 10:**
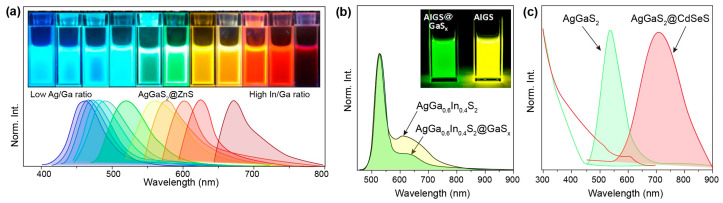
(**a**) Photographs and photoluminescence spectra of AgGaS_2_@ZnS nanocrystals with varied Ag/Ga or In/Ga ratios. Reproduced with permission [101]. Copyright 2020, American Chemical Society. (**b**) Photoluminescence spectra of AgGa_0.6_In_0.4_S_2_ and AgGa_0.6_In_0.4_S_2_@GaS_x_ nanocrystals. Reproduced with permission [103]. Copyright 2018, American Chemical Society. (**c**) Absorption and photoluminescence spectra of AgGaS_2_ and AgGaS_2_@CdSeS nanocrystals, respectively. Reproduced with permission [105]. Copyright 2021, Elsevier Ltd.

The growth of an epitaxial shell on the surface of nanocrystals is a common approach for achieving surface passivation as well as emission tuning, depending on the alignment between core and shell band structures [109,111,112]. The electronic band in a type-I structure is characterized by a straddling band alignment, in which the wider-bandgap shell confines the wave function of the electron–hole pair within the AgGaS_2_ region. ZnS is the most popular shell material for passivating AgGaS_2_ nanocrystals due to its large bulk bandgap, proper band alignment, and superior stability. Bai and coworkers developed a one-pot strategy for the synthesis of AgGaS_2_@ZnS core–shell nanocrystals without prior purification of the as-synthesized AgGaS_2_ core nanocrystals [101]. Structural characterization was performed to confirm the AgGaS_2_@ZnS with a designed core–shell configuration. The energy bandgap of the AgGaS_2_@ZnS core–shell nanocrystals was effectively tunable from 2.98 to 2.83 eV by controlling their non-stoichiometry (Figure 10a). In addition, it could further continuously decrease to 1.90 eV in AgGa_x_In_1−x_S_2_@ZnS core–shell nanocrystals in conjunction with the In alloying strategy. Benefitting from surface passivation, the resultant core–shell nanocrystals showed largely improved quantum yields of ~30% for LED application.

In addition to the use of ZnS as a shell component, researchers have found that GaS_x_ and CdSeS can also be employed as shells for different purposes. For example, when AgIn_x_Ga_1−x_S_2_ nanocrystals were coated with GaS_x_, the original broad defect-site emission peak was drastically and selectively suppressed, leading to an increase in the quantum yield of the band-edge emission peak (Figure 10b) [103]. The optimal quantum yield of AgIn_x_Ga_1−x_S_2_@GaS_x_ core–shell nanocrystals was 28%, with green band-edge emission at 530 nm and a full width at half-maximum of 181 meV (41 nm). After GaS_x_ coating, the intensity of the defect-site emission was remarkably reduced to less than 15% of the intensity of the corresponding bandgap emission. CdSeS shelling generally leads to inducing a type-II band structure. A red shift of the photoluminescence peak was observed for the AgGaS_2_@CdSeS core–shell nanocrystals, the emission of which was ascribed to the recombination of delocalized electrons in the CdSeS shell and localized holes within the AgGaS_2_ core QDs (Figure 10c) [105]. Such AgGaS_2_@CdSeS core–shell nanocrystals with charge carrier separation features are useful in photovoltaic applications [113]. In another work, Wang and coworkers reported efficient blue LEDs using AgGaS_2_@GaS_x_ as the light-emitting layer, demonstrating the potential of AgGaS_2_ quantum dots as a high-quality blue light-emitting material [88].

One may have noticed that during shell growth, a band shift in both absorption and emission spectra in comparison to those of the AgGaS_2_ core nanocrystals is always observed, implying that the practical outcome of core–shell engineering may result from complex interplays such as etching, cation exchange, and alloying [114]. This phenomenon has also been widely reported in other ternary chalcopyrites like CuInS_2_ [115]. In addition, either for lighting or for photovoltaic applications, there is a trade-off between shell thickness and performance. Current demand requires core–shell nanocrystals with sufficient shell thickness but suppressed alloying. In this regard, the development of low-temperature deposition techniques may be a potential option. In addition, stoichiometry control is another manner for optical tuning in other ternary systems such as CuInS_2_ [116,117]; however, this technique has not been well studied for AgGaS_2_. There are few data concerning the role of the Ag/Ga ratio in optical tuning; 1:8 is adopted in some previous reports [83,100]. Due to the fact that a higher degree of Ag deficiency in AgGaS_2_ can lower its valence band maximum, since it is composed of the hybrid orbitals Ag 4d and S 3p, the study of stoichiometry control, especially the Ag/Ga ratio, deserves special attention in the Ag–Ga–S system in future work.

## 6. Conclusions

This review documents the development of bulk and nanocrystalline AgGaS_2_ as well as their derivatives from the perspective of synthesis, NLO response, and optical tuning. With significant advancements made over the past decade, the study of AgGaS_2_ has stimulated the further exploitation of a series of diamond-like AgGaS_2_ derivatives with larger bandgaps, higher LIDTs, or stronger SHG coefficients, expanding the toolbox of infrared NLO materials. In addition, AgGaS_2_ and derived nanocrystals with suitable sizes and good mono-dispersion can be obtained through direct synthetic control or post-synthesis cation exchange. Through band intermixing, alloyed nanocrystals also provide more possibilities for tuning their absorption and emission features. Despite the encouraging achievements, there are still challenges that need to be addressed before AgGaS_2_ and its derivatives can gain equal popularity to other well-established nanocrystals such as chalcopyrite CuInS_2_, II-VI/III-V materials, and perovskites. Further research is needed to address issues related to synthesis scalability, long-term stability, and understanding the fundamental properties and behaviors of these materials.

First, historically, diamond-like compounds were studied extensively, but their NLO properties were largely overlooked until recent years. To fully exploit the potential of these materials, it is necessary to conduct a comprehensive structural identification of the available material systems. One effective approach is the incorporation of certain cations that exhibit the second-order Jahn–Teller effect, such as d^0^ transition-metal cations (e.g., Au^+^, Hf^4+^, Nb^5+^), and main-group cations that possess lone electron pairs (e.g., Pb^2+^, Sn^2+^, Bi^3+^, Sb^3+^, Te^4+^). These cations can introduce structural distortions and create favorable conditions for strong SHG responses. Furthermore, the modification of these compounds through appropriate alkali metal doping can be a promising strategy to increase the bandgap energy. This can lead to improved NLO properties, as well as enhanced stability and practicality for various applications. Advancements in high-throughput theoretical calculations have also been valuable in the search for high-quality NLO materials. These calculations can predict the NLO properties of materials based on their crystal structures and electronic properties, providing valuable guidance for experimental synthesis and characterization.

Second, the luminescence mechanism of AgGaS_2_-based nanocrystals requires further investigation and clarification. Some researchers have recorded large FWHMs of AgGaS_2_ nanocrystals (150–200 nm) [97] and attributed their luminescence to donor–acceptor pair recombination or free-to-bound recombination, which have been widely adopted for explaining the electronic transition of CuInS_2_. An ultra-large Stokes shift of 550–650 meV has also been observed in AgGaS_2_ nanocrystals [100], which is even more significant compared to the intensively studied CuInS_2_ nanocrystals. To understand the luminescence mechanism of AgGaS_2_ nanocrystals, several aspects should be addressed. On the one hand, if the donor–acceptor pair model is applicable, it is important to determine the locations of the donor and acceptor levels and investigate the dynamics of the transition using ultrafast spectroscopy in combination with theoretical modeling. This can provide insights into the energy levels involved in the luminescence processes. On the other hand, if the free-to-bound model is applicable, it is necessary to determine whether the recombination occurs from localized electronic defects to delocalized valence band (VB) states or from delocalized conduction band (CB) states to localized electronic defects. Understanding this process can shed light on the nature of the electronic defects and the fine structure of the electronic bands involved in the luminescence. To gain a deeper understanding of luminescence mechanisms, it would be beneficial to conduct systematic studies that investigate the excitation-dependent properties of emission. By carefully examining the emission spectra under different excitation conditions, valuable information about the intra-bandgap states and their involvement in the luminescence can be obtained.

Third, bulk AgGaS_2_ and AgGaS_2_ nanocrystals are expected to excel in novel applications. In the case of bulk AgGaS_2_, it is expected to excel in quantum imaging and sensing techniques in the mid-infrared range. Nondegenerate photon-pair generation through spontaneous parametric downconversion has been observed in AgGaS_2_ crystals. Researchers have reported the generation of idler photons in the mid-infrared spectral range above 6 µm in wavelength, accompanied by signal photons in the visible range [118]. This capability is significant considering the availability of single-photon detectors developed for infrared wavelengths up to 10 µm. The broad spectral bandwidth of photon-pair generation in AgGaS_2_ adds to its potential for various applications in quantum imaging and sensing [119]. On the other hand, AgGaS_2_ nanocrystals offer blue–green emission due to their suitable bandgap energy. This fluorescence in the range below 460 nm makes them suitable for a variety of lighting applications. Additionally, AgGaS_2_ nanocrystals can be used as shell materials in systems with smaller bandgaps, such as CuInS_2_ nanocrystals. By coating CuInS_2_ nanocrystals with AgGaS_2_, the resulting hybrid structures can exhibit multifunctional properties and enable a wider range of applications. Wet-chemical synthesis approaches for AgGaS_2_ nanocrystals hold promise for achieving multifunctionality and tailoring their properties for specific applications. These advancements open up exciting possibilities for the practical utilization of AgGaS_2_ in various fields.

Last but not the least, the environmental impact of the extraction of raw materials and the preparation of AgGaS_2_ and its derivatives deserves further investigation. As rare but critical metal elements, Ga and Ag have been widely used in electronic and optical devices in many forms besides AgGaS_2_, such as conductive silver pastes (for electrodes), copper–indium–gallium–chalcogenide materials (CIGS, for photovoltaic absorbers), silver sulfide (AgS, for near-infrared quantum dots), and so on. Certainly, the recovery and recycling of silver and gallium should be sufficiently prioritized to minimize their environmental impact.

Overall, the research on bulk and nanocrystal AgGaS_2_ driven by both scientific curiosity and application prospects is undoubtedly flourishing. Considering the rapid progress and growing interest in AgGaS_2_, it is reasonable to anticipate that this material will continue to play a significant and irreplaceable role in various fields, pushing the boundaries of NLO, optoelectronics, and beyond.

## Figures and Tables

**Table 1:** NLO parameters of AgGaS_2_ and typical AgGaS_2_-derived crystals with diamond-like structures.

Type	Compound	Space Group	Eg (eV)	Band Types	IR Cutoff (μm)	Δn@1.06 μm	SHG *d*_ij_ (pm/V)	LIDT (×AgGaS_2_)	Ref
Commercially available	AgGaS_2_	*I*-42*d*	2.7	Direct	0.45–13	0.053	*d*_36_ = 13.9	1.0	[34]
	AgGaSe_2_	*I*-42*d*	1.8	Direct	0.76–17	N.A.	*d*_36_ = 33.0	N.A.	[46]
	ZnGeP_2_	*I*-42*d*	1.74	Direct	0.74–12	0.046	*d*_eff_ = 75.0	N.A.	[47]
Bandgap modification	NaGaS_2_	*I*-42*d*	3.9	Direct	0.31–13.3	0.094	*d*_36_ = 13.2	1.1	[48]
	Li_2_ZnSiS_4_	*P*na2_1_	3.9	Direct	25	N.A.	*d*_33_ = 18.9	10.0	[49]
	[Ba_4_C_l2_] [ZnGa_4_S_10_]	*I*-4	3.8	Direct	0.29–13.7	0.012@2.05 μm	*d*_14_ = 14.9	51.0	[50]
	LiGaS_2_	*P*na2_1_	3.7	Direct	11.6	0.014	*d*_33_ = 11.2	11.0	[51]
	Ba_6_Zn_7_Ga_2_S_16_	*R*3	3.5	Direct	N.A.	0.036	*d*_11_ = 6.1	28.0	[52]
	AgGaS_2_:Li	*I*-42*d*	3.4	Direct	N.A.	N.A.	*d*_36_ = 20.6	8.6	[53]
	Na_2_ZnGe_2_S_6_	*Cc*	3.2	Direct	0.38–22	0.026	*d*_33_ = −5.3	6.0	[54]
	Na_2_Ga_2_GeS_6_	*Fdd*2	3.1	Direct	N.A.	N.A.	*d*_eff_ = 11.2	18.1	[55]
	Ba_2_Ga_8_GeS_16_	*P*6_3_*mc*	3.0	Direct	0.42–20	N.A.	*d*_eff_ = 26.0	22.0	
	CuZnPS_4_	*I*-42m	3.0	Indirect	16.5	0.07	*d*_14_ = 15.9	6.0	
SHG enhancement	Na_2_Ga_2_SnS_6_	*Fdd*2	2.7	Direct	N.A.	N.A.	*d*_eff_ = 13.1	17.9	[55]
	AgHgPS_4_	*P*n	2.6	Indirect	12	0.11@2.09 μm	*d*_11_ = −31.1	N.A.	[56]
	Li_2_HgGeSe_4_	*P*na*2*_1_	1.7	Direct	N.A.	0.042–0.074	*d*_33_ = −90.2	3.0	[57]
	Li_2_HgSnSe_4_	*P*na*2*_1_	1.6	Direct	N.A.		*d*_33_ = −104	3.5	
	Na_2_Hg_3_Ge_2_Se_8_	*P*-4*c*2	1.3	Direct	N.A.	0.096–0.172	*d*_36_ = 87.6	4.5	
	Na_2_Hg_3_Sn_2_Se_8_	*P*-4*c*2	1.2	Direct	N.A.		*d*_36_ = 96.4	3.0	

**Table 2:** A conclusion of wet-chemistry-synthesized AgGaS_2_ and typical AgGaS_2_-derived nanocrystals.

Nanocrystals	Phase	Precursors	Size (nm)	Eg (eV)	Emission Range (nm)	Ref
AgGaS_2_	Tetragonal	AgCl, Ga, and S	5.0–7.0	N.A.	N.A.	[92]
AgGaS_2_	Tetragonal	AgCl, GaCl_3_, and thiourea	5.0	N.A.	446	[93]
AgGaS_2_	Tetragonal	Ag-oleate, Ga(acac)_3_, S	13.0	2.6–2.7	475	[82]
AgGaS_2_	Orthorhombic	AgS_2_CN(C_2_H_5_)_2_, Ga(S_2_CN(C_2_H_5_)_2_)_3_	16.3–19.1	2.7	N.A.	[94]
AgGaS_2_	Monoclinic	AgNO_3_, Ga(acac)_3_, and S	3–5.6	N.A.	460, 650	[95]
AgGaS_2_	Orthorhombic, rhombohedral	AgNO_3_, GaCl_3_, Na_2_S,	2.0	N.A.	N.A.	[96]
AgGaS_2_: In	Tetragonal	AgInyGa1-y(S_2_CN(C_2_H_5_)_2_)_4_	4–5	N.A.	550–750	[97]
AgGaS_2_: In	Tetragonal	AgInS_2_, Ga(NO_3_)_3_	4.2	N.A.	550	[98]
AgGaS_2_: Zn	Tetragonal	AgNO_3_, Ga(acac)_3_, ZnSt_2_, and S	4.2	N.A.	470–510	[83]
AgGaS_2_: Se	Orthorhombic, tetragonal	AgNO_3_, GaSt_3_, InSt_3_, Se, thiourea	10–18	1.9–2.8	N.A.	[99]
AgGaS_2_: In@ZnS	Tetragonal	AgI, Ga(acac)_3_ In(Ac)_3_, and S	5–5.3	2.4–2.9	515–570	[100]
AgGaS_2_: Zn@ZnS	Tetragonal	AgI, Ga(acac)_3_ ZnCl_2_, and S	5–5.3	2.9–3.1	450–515	
AgGaS_2_: In@ZnS	Tetragonal	AgI, Ga(acac)_3_ ZnSt_2_, and S	5.0	1.9–2.8	460–670	[101]
AgGaS_2_: In@ZnS	Tetragonal	AgNO_3_, Ga(Ac)_3_, In(Ac)_3_, and S	3.8	N.A.	560–600	[102]
AgGaS_2_: In@GaS_x_	Tetragonal	AgAc, Ga(acac)_3_, In(acac)_3_, and S	2.9–4.5	2.1–2.6	500–600	[103]
AgGaS_2_: In@GaS_x_	Tetragonal	AgAc, In(Ac)_3_, Ga(S_2_CN(C_2_H_5_)_2_)_3_	4.3	2.1–2.6	498–602	[104]
AgGaS_2_@CdSeS	Tetragonal	AgI, Ga(acac)_3_, and S	5.1 ± 0.6	1.97	710	[105]

## Data Availability

The data are available upon request.

## Abbreviations

The following abbreviations are used in this manuscript:

NLO	Nonlinear optics
OPO	Optical parametric oscillation
SHG	Second harmonic generation
LIDT	Laser-induced damage threshold
SOJT	Second-order Jahn–Teller
VEC	Valence electron concentration
ICP-AES	Inductively coupled plasma atomic emission spectroscopy
FWHM	Full width at half-maximum
LEDs	Light-emitting diodes
